# Assessment of visual and auditory evoked potentials in multiple sclerosis patients with and without fatigue

**DOI:** 10.1007/s10072-014-1953-8

**Published:** 2014-09-21

**Authors:** Anna Pokryszko-Dragan, Malgorzata Bilinska, Ewa Gruszka, Elżbieta Kusinska, Ryszard Podemski

**Affiliations:** Department of Neurology, Medical University of Wroclaw, Borowska 213, 50-556 Wrocław, Poland

**Keywords:** Multiple sclerosis, Fatigue, Evoked potentials

## Abstract

The aim of the study was to evaluate visual and brainstem auditory evoked potentials (VEP, BAEP) in multiple sclerosis (MS) patients with regards to fatigue and disease-related variables. The study comprised 86 MS patients and 40 controls. Fatigue was assessed using the Fatigue Severity Scale (FSS/FSS-5) and the Modified Fatigue Impact Scale (MFIS). Latencies and amplitudes of the P100 component of VEP and the I–V components of BAEP were analyzed. The results of EP were compared between non-fatigued, moderately and severely fatigued MS patients and controls. P100 latency was increased and amplitude decreased in moderately and severely fatigued MS subjects. The latency of the V component of BAEP and interlatencies I-III-V were increased in severely fatigued patients. The amplitude of the V component was lowered in fatigued patients. VEP and BAEP abnormalities were usually one-sided. Interocular P100 latency difference tended to correlate with FSS/FSS-5. The parameters of VEP and BAEP correlated with functional system scores but not with MS duration, overall degree of disability or its progression over time. Significant, usually asymmetrical VEP and BAEP abnormalities were found in fatigued MS patients, with no relationships to disease-related variables. EP may be considered an electrophysiological marker of fatigue in MS patients.

## Introduction

Fatigue is commonly reported by patients with multiple sclerosis and has a profound impact upon their daily activities and quality of life. Although often discussed in recent years, the origin of fatigue remains unclear. Evaluation of fatigue also brings about difficulties because of its lack of the objective biomarkers [1, 2]. According to some theories, the background of fatigue is associated with disturbed bioelectrical neuronal activity due to demyelination and axonal loss [3, 4]. Studies with the use of motor evoked potentials and electroencephalography event-related desynchronization have indeed shown decreased neuronal excitability and frequency-dependent conduction block in fatigued MS patients [5–8]. Visual and auditory evoked potentials are regarded as useful tools for recognizing and monitoring damage to central nervous system ascending pathways in the course of MS. However, these methods have not been used so far in studies on MS fatigue, investigating its origin and methods of its evaluation.

The aim of our study was to assess visual and brainstem auditory evoked potentials (BAEP) in MS patients with regard to the presence and severity of fatigue, considering also the impact of other disease-related variables.

## Materials and methods

The study comprised 86 patients with MS (24 men and 62 women, aged 19–60 years, mean 39.55) who were under the care of the outpatient MS clinic, Department of Neurology, Medical University of Wroclaw. All the patients met the McDonald’s criteria [9] of clinically definite MS. None of the patients had concomitant diseases known to cause fatigue or to affect parameters of visual and auditory evoked potentials in their history. 62 patients had never been treated with disease-modifying agents. 24 subjects had undergone treatment with interferon beta or glatiramer acetate for 1–3 years, but treatment had ceased at least 6 months prior to their inclusion in this study (in 15 patients because of their transition into secondary progressive phase of MS; nine subjects resigned from the treatment due to its side effects or for personal reasons). None of the patients were being treated with chronic immune-suppression. A washout period of at least 4 weeks was maintained between inclusion in the study and tapering treatment with corticosteroids due to the most recent MS relapse.

The control group consisted of 40 healthy volunteers, who were matched for age and gender to the MS patients (12 men, 28 women, aged 23–60 years, mean 38.8).

All the subjects gave their informed consent to participate in the study and the project was approved by the Bioethical Committee at the Medical University of Wroclaw.

The patients underwent a neurological examination and their disability was assessed using the Expanded Disability Status Scale (EDSS) [10], with visual and brainstem functional systems (FS) scores separated for further analysis. On the basis of medical records, the duration of the disease was defined and the index of disability progression [Multiple Sclerosis Severity Scale (MSSS)] calculated [11]; the history of optic neuritis or loss of hearing was also determined.

### Assessment of fatigue

The level of fatigue in MS patients was evaluated using self-assessment questionnaires based on the Fatigue Severity Scale (FSS) [12] and the Modified Fatigue Impact Scale (MFIS) [13], with the results of FSS re-evaluated using the Rasch analysis applied by Mills et al. [14] (FSS-5). On the basis of FSS/FSS-5 results, the patients were divided into three subgroups: without fatigue (subgroup I, FSS/FSS-5 <3.5), with moderate (subgroup II, FSS/FSS-5 = 3.5–5.5) or severe fatigue (subgroup III, FSS/FSS-5 >5.5).

### Evoked potentials

Visual evoked potentials (VEP) were performed by using a black and white checker board pattern on a screen, with a checker size of 36 cm^2^ and the frequency of pattern reversing being 1.9 Hz. The subjects were sitting in the distance of 1 m from the screen, with the angle of vision 2°9′. The stimuli were presented uni-ocularly. An active recording electrode was attached to the scalp on the midline at the occipital region (Oz according to the 10–20 system), the reference electrode was placed on the midline frontal point (Fz) and the ground electrode on the forearm. Ag/AgCl surface electrodes were used and their impedance was maintained below 5 k Ohm. The responses were analyzed with a Nicolet 1000 Viking Quest, with a 1–30 Hz bandpass filter and a sweep time of 500 ms. A hundred responses were averaged in each run and two runs were performed for each eye. For each subject, the latency and amplitude (“peak to peak”) of the P100 component were determined for each eye, as well as relative P100 latency (interocular latency difference).

BAEP were performed with the use of stimuli presented to each ear separately via earphones. Auditory stimuli were clicks of a duration of 0.1 ms, frequency 20.3 Hz and an intensity 65 dB higher than the hearing threshold initially established for each subject. A recording electrode was attached to the earlobe on the side of stimulation, with the reference electrode placed on the vertex (Fz) and the ground electrode on the forearm. Ag/AgCl surface electrodes were used and their impedance was maintained below 5 k Ohm. The responses were analyzed with a Nicolet 1000 Viking Quest, with a 150–3,000 Hz bandpass filter and a sweep time of 10 ms. Two hundred responses were averaged in each run and two runs were performed for each ear. Latencies and amplitudes (“peak to peak”) were determined for components I, III and V, as well as interpeak latencies I–III, III–V, I–V and the proportion of amplitudes I/V.

### Statistical analysis

Mean and median values with standard deviations were calculated for all the analyzed variables. The EP parameters obtained from the whole group of MS patients and subgroups I, II and III were compared with those from the controls, and the results were also compared between subgroups I, II and III with the use of post hoc test (Scheffe test) and then analysis of variance (ANOVA), alternatively using the Kruskal–Wallis test, when the variances in groups were not homogeneous (the homogeneity of variance was determined by the Bartelett’s test) or if the number of cases was too small. Relation between continuous fatigue measures and continuous EP parameters was assessed using correlation analysis and Pearson correlation coefficients were calculated. Relation between continuous fatigue measures and categorized parameters (visual FS, brainstem FS) was assessed using correlation analysis and Spearman correlation coefficients were calculated. Multiple regression analysis was used to check the impact of age and MS-related variables upon correlations between fatigue measures and EP results. *p* < 0.05 was regarded as statistically significant and *p* < 0.07 sufficient to observe trends. The statistical analysis was performed using EPIINFO ver. 3.5.2 software.

## Results

On the basis of FSS/FSS-5 results, 29 patients (8 men, 21 women) were allocated to subgroup I (non-fatigued), 31 patients (7 men, 24 women) to subgroup II (moderately fatigued) and 26 (8 men, 18 women) to subgroup III (severely fatigued). No significant differences in terms of age or gender were found either between these groups, or between each of them and the healthy controls.

The results of MFIS on the whole group of patients ranged from 4 to 64 (mean 36.3). There was a significant correlation between MFIS results and the age of the MS patients (*R* = 0.24, *p* = 0.02). No such correlations were found for FSS/FSS-5 results.

In MS patients the duration of the disease was 1–30 years (mean 8.57), EDSS 1–6.5 (mean 3.03) and MSSS 1.1–8.8 (mean 4.4). Visual FS scores ranged from 0 to 3 (mean 0.8), and so did Brainstem FS scores (mean 1.2). 42 patients had a history of optic neuritis, while none experienced loss of hearing during MS relapse. Patients with or without the history of optic neuritis did not differ significantly in the mean values of FSS/FSS-5 (4.63 vs 4.02, *p* = 0.1) or MFIS (39.1 vs 33.7, *p* = 0.12). FSS/FSS-5 and MFIS correlated significantly with EDSS, visual and brainstem FS (Table 1).

**Table 1:** Correlations between fatigue measures (FSS/FSS-5, MFIS) and degree of disability (EDSS, visual FS, brainstem FS); *R* Spearman’s correlation coefficient

	EDSS	Visual FS	Brainstem FS
FSS/FSS-5	*R* = 0.48	*R* = 0.3	*R* = 0.26
	*p* = 0.00001	*p* = 0.006	*p* = 0.018
MFIS	*R* = 0.46	*R* = 0.32	*R* = 0.24
	*p* = 0.0001	*p* = 0.003	*p* = 0.025

The mean duration of the disease was longer in subgroup III in comparison with subgroup I (11.56 vs 5.38 years, *p* = 0.003). The mean EDSS score was higher in subgroups II and III in comparison with subgroup I (2.98 vs 4.08 vs 2.12, *p* = 0.027 and 0.0000001, respectively). The mean MSSS score was higher in subgroup III in comparison with subgroups II and I (5.32 vs 4.16 vs 3.86, *p* = 0.047 and 0.014, respectively).

The mean latency of the P100 component of VEP for both eyes was significantly longer in MS patients than in the controls, and so was the mean relative P100 latency (interocular latency difference). The mean P100 amplitude was significantly lower for MS patients than in controls, but only for the left eye (Table 2).

**Table 2:** Parameters of the P100 component of visual evoked potentials in the controls, whole MS group and subgroups of MS patients: non-fatigued (I), moderately (II) and severely (III) fatigued

	Controls (*n* = 40)	MS patients (*n* = 86)		MS subgroup I (*n* = 29)		MS subgroup II (*n* = 31)		MS subgroup III (*n* = 26)	
L: P100 latency (ms)									
Mean	101.8	117.3	***p *** **(MS-contr)** **=** **0.00001**	113.9	***p*** ** (I-contr)** **=** **0.00001**	118.3	***p*** ** (II-contr)** **=** **0.00001**	119.8	***p*** ** (III-contr)** **=** **0.00001**
SD	5.0	21.6		14.9		16.1	*p* (I–II) = 0.28	31.9	*p* (I–III) = 0.38
									*p* (II–III) = 0.82
R: P100 latency (ms)									
Mean	102.2	119.9	***p*** ** (MS-contr)** **=** **0.00001**	113.8	***p*** ** (I-contr)** **=** **0.00001**	121.1	***p *** **(II-contr)** **=** **0.00001**	125.1	***p*** ** (III-contr)** **=** **0.00001**
SD	4.7	15.8		12.9		14.6	***p*** **(I**–**II)** **=** **0.02**	18.4	***p*** ** (I**–**III)** **=** **0.006**
									*p* (II–III) = 0.2
Relative P100 latency:									
Mean	2.14	9.95	***p*** ** (MS-contr)** **=** **0.00001**	6.49	***p*** ** (I-contr)** **=** **0.0002**	12.15	***p*** ** (II-contr)** **=** **0.00001**	11.16	***p*** ** (III-contr)** **=** **0.00001**
SD	1.96	11.13		6.63		13.13	*p* (I–II) = 0.15	11.96	***p*** ** (I**–**III)** **=** **0.038**
									*p* (II–III) = 0.89
L: P100 amplitude (µV)									
Mean	11.4	9.39	***p*** ** (MS-contr)** **=** **0.027**	9.72	*p* (I-contr) = 0.15	9.34	*p* (II-contr) = 0.057	9.06	*p* (III-contr) = 0.052
Median	4.5	4.92		5.25		4.63	*p* (I–II) = 0.49	5.08	*p* (I–III) = 0.65
SD									*p* (II–III) = 0.83
R: P100 amplitude (µV)									
Mean	10.6	8.97	*p *(MS-contr) = 0.075	8.96	*p *(I-contr) = 0.15	9.25	*p* (II-contr) = 0.22	8.63	*p* (III-contr) = 0.097
SD	4.4	5.05		8.43		7.92	*p *(I–II) = 0.83	5.12	***p*** ** (I**–**III)** **=** **0.012**
				5.07		5.13			*p* (II–III) = 0.65
Relative P100 amplitude:									
Mean	0.97	1.02	*p* (MS-contr) = 0.47	1.07	*p* (I-contr) = 0.55	1.02	*p* (II-contr) = 0.46	0.98	*p (*III-contr) = 0.68
SD	0.22	0.4		0.56		0.28	*p* (I–II) = 0.53	0.38	*p* (I–III) = 0.50
									*p* (II–III) = 0.36

Bold values are statistically significant at (*p* < 0.05)

*L* left eye, *R* right eye)

P100 latency for the right eye was significantly longer in subgroups II and III than in subgroup I, and relative P100 latency was significantly longer in subgroup III than in subgroup I (Table 2). The amplitude of P100 for the left eye was lower in subgroups II and III than in the controls, and for the right eye—lower in subgroup III than in subgroup I (Table 1). No significant correlations were found between the summated values of VEP latency and amplitude and FSS/FSS-5 or MFIS. The relative latency of P100 tended to correlate positively with FSS/FSS-5 (*R* = 0.26, *p* = 0.07). There was a significant correlation between summated VEP amplitude and Visual FS (*R* = −0.36, *p* = 0.0006) and a correlation on the edge of significance between summated VEP latency and Visual FS (*R* = 0.21, *p* = 0.05). No correlations were found between VEP parameters and other clinical MS-related variables (duration of the disease, EDSS or MSSS).

The mean latencies of I, III and V components of BAEP on both sides did not differ significantly between MS patients and controls. Mean interlatencies I–III on the left side, III–V on the right side and I–V on both sides were significantly longer in patients than in the controls. The mean amplitude of the V component was significantly lower in MS patients than in the controls, but only on the right side (Table 3).

**Table 3:** Parameters of brainstem auditory evoked potentials in the controls, whole MS group and subgroups of MS patients: non-fatigued (I), moderately (II) and severely (III) fatigued

	Controls (*n* = 40)	MS patients (*n* = 86)		MS subgroup I (*n* = 29)		MS subgroup II (*n* = 31)		MS subgroup III (*n* = 26)	
**L:** latency I									
Mean	1.69	1.68	*p* (MS-contr) = 0.67	1.66	*p* (I-contr) = 0.28	1.69	*p* (II-contr) = 0.98	1.70	*p* (III-contr) = 0.97
SD	0.13	0.12		0.1		0.13	*p* (I–II) = 0.30	0.12	*p* (I–III) = 0.28
									*p* (II–III) = 0.99
Latency III									
Mean	3.83	3.87	*p* (MS-contr)=0.24	3.87	*p* (I-contr) = 0.26	3.860.16	*p* (II-contr) = 0.14	3.91	*p* (III-contr) = 0.06
SD	0.12	0.17		0.17			*p* (I–II) = 0.84	0.20	*p* (I–III) = 0.43
									*p* (II–III) = 0.30
Latency V									
Mean	5.7	5.77	*p* (MS-contr)=0.53	5.86	*p* (I-contr) = 0.23	5.78	*p* (II-contr) = 0.92	5.65	***p*** **(III-contr)** **=** **0.049**
SD	0.21	0.73		0.44		0.24	*p* (I–II) = 0.83	1.23	*p* (I–III) = 0.39
									*p* (II–III) = 0.30
Interlat. I–III									
Mean	2.13	2.19	***p*** **(MS-contr)** **=** **0.046**	2.2	*p* (I-contr) = 0.053	2.16	*p* (II-contr) = 0.39	2.21	*p* (III-contr) = 0.054
SD	0.14	0.16		0.17		0.16	*p* (I–II) = 0.31	0.17	*p* (I–III) = 0.63
									*p* (II–III) = 0.11
Interlat. III–V									
Mean	1.87	1.96	*p* (MS-contr) = 0.072	1.99	*p* (I-contr) = 0.33	1.93	*p* (II-contr) = 0.24	1.97	*p* (III-contr) = 0.16
SD	0.17	0.30		0.38		0.23	*p* (I–II) = 0.42	0.27	*p* (I–III) = 0.65
									*p* (II–III) = 0.63
Interlat. I–V									
Mean	4.0	4.15	***p*** **(MS-contr)** **=** **0.013**	4.2	*p* (I-contr) = 0.057	4.09	*p* (II-contr) = 0.098	4.19	***p*** **(III-contr)** **=** **0.025**
SD	0.19	0.34		0.43		0.25	*p* (I–II) = 0.69	0.34	*p* (I–III) = 0.64
									*p* (II–III) = 0.36
Amp. I									
Mean	0.3	0.32	*p* (MS-contr) = 0.58	0.32	*p* (I-contr) = 0.56	0.30	*p* (II-contr) = 0.93	0.33	*p* (III-contr) = 0.43
SD	0.1	0.14		0.12		0.14	*p* (I–II) = 0.69	0.15	*p* (I–III) = 0.81
									*p* (II–III) = 0.58
Amp. V									
Mean	0.44	0.4	*p* (MS-contr) = 0.33	0.39	*p* (I-contr) = 0.19	0.44	*p* (II-contr) = 0.92	0.37	*p* (III-contr) = 0.43
SD	0.16	0.24		0.17		0.18	*p* (I–II) = 0.28	0.34	*p* (I–III) = 0.85
									*p* (II–III) = 0.37
**R:** latency I									
Mean	1.7	1.72	*p* (MS-contr) = 0.85	1.73	*p* (I-contr) = 0.61	1.73	*p* (II-contr) = 0.69	1.69	*p* (III-contr) = 0.64
SD	0.14	0.19		0.19		0.18	*p* (I–II) = 0.91	0.21	*p* (I–III) = 0.47
									*p* (II–III) = 0.51
Latency III									
Mean	3.85	3.88	*p* (MS-contr) = 0.63	3.850.42	*p* (I-contr) = 0.83	3.89	*p* (II-contr) = 0.44	3.89	*p* (III-contr) = 0.46
SD	0.16	0.33				0.28	*p* (I–II) = 0.65	0.29	*p* (I–III) = 0.47
									*p* (II–III) = 0.99
Latency V									
Mean	5.73	5.81	*p* (MS-contr) = 0.52	5.78	*p* (I-contr) = 0.80	5.91	***p*** **(II-contr)** **=** **0.03**	5.71	*p* (III-contr) = 0.11
SD	0.23	0.81		0.23		0.48	*p* (I–II) = 0.32	1.28	*p* (I–III) = 0.79
									*p* (II–III) = 0.42
Interlat. I–III									
Mean	2.14	2.18	*p* (MS-contr) = 0.26	2.18	*p* (I-contr) = 0.31	2.16	*p* (II-contr) = 0.61	2.20	*p* (III-contr) = 0.25
SD	0.12	0.19		0.16		0.21	*p* (I–II) = 0.76	0.20	*p* (I–III) = 0.78
									*p* (II–III) = 0.31
Interlat. III–V									
Mean	1.87	2.0	***p*** **(M**S**-contr)** **=** **0.013**	1.95	*p* (I-contr) = 0.21	2.02	***p*** **(II-contr)** **=** **0.005**	2.05	***p*** **(III-contr)** **=** **0.04**
SD	0.18	0.3		0.28		0.25	*p* (I–II) = 0.26	0.37	*p* (I–III) = 0.29
									*p* (II–III) = 0.99
Interlat. I–V									
Mean	4.02	4.19	***p*** **(MS-contr)** **=** **0.042**	4.12	*p* (I-contr) = 0.52	4.18	*p* (II-contr) = 0.058	4.26	***p*** **(III-contr)** **=** **0.025**
SD	0.2	0.39		0.35		0.39	*p* (I–II) = 0.52	0.43	*p* (I–III) = 0.19
									*p* (II–III) = 0.56
Amp. I									
Mean	0.3	0.29	*p* (MS-contr) = 0.68	0.32	*p* (I-contr) = 0.63	0.28	*p* (II-contr) = 0.35	0.27	*p* (III-contr) = 0.34
SD	0.1	0.18		0.23		0.13	*p* (I–II) = 0.37	0.15	*p* (I–III) = 0.38
									*p* (II–III) = 0.92
Amp. V									
Mean	0.46	0.37	***p*** **(MS-contr)** **=** **0.025**	0.420.24	*p* (I-ontr) = 0.52	0.37	*p* (II-contr) = 0.054	0.31	***p*** **(III-contr)** **=** **0.003**
SD	0.19	0.2				0.19	*p* (I–II) = 0.31	0.17	*p* (I–III) = 0.057
									*p* (II–III) = 0.28

Bold values are statistically significant at (*p* < 0.05)

*L* left ear, *R* right ear, *interlat.* interlatency, *amp* amplitude

The latency of the V component of BAEP, and interlatency I–V for the left ear as well as interlatencies III–V and I–V for the right ear were significantly longer in subgroup III than in the controls. In subgroup II, the latency of the V component and interlatencies III–V, and I–V for the right ear were significantly longer than in the controls (Table 3). The amplitude of the V component for the right ear was significantly lower in subgroups II and III than in the controls (Table 3). No correlations were found between the BAEP parameters (one-sided or summated values) and fatigue measures (FSS/FSS-5, MFIS). Among the BAEP parameters, interlatencies III–V and I–V showed significant positive correlations with MSSS (III–V: *R* = 0.28, *p* = 0.009; *R* = 0.25, *p* = 0.024; I–V: *R* = 0.25, *p* = 0.021; *R* = 0.2, *p* = 0.05; for left and right side, respectively). Summated latencies of BAEP components correlated significantly with Brainstem FS (I: *R* = 0.23, *p* = 0.04; III: *R* = 0.23, *p* = 0.03; V: *R* = 0.36, *p* = 0.0008) and so did summated amplitude of V component (*R* = 0.23, *p* = 0.03). No other correlations were found between BAEP parameters and remaining clinical MS-related variables (duration of the disease, EDSS or MSSS).

## Discussion

The importance of evoked potentials (EP) in the diagnosis of MS has decreased in the last decade as magnetic resonance has become the main diagnostic tool supporting clinical assessment. However, EP abnormalities are still regarded as good electrophysiological markers of disease progression and their prognostic value in the early stages of MS gains increasing attention [15–17]. Non-invasiveness and the availability of EP also encourage their use in clinical practice. Fatigue constitutes an important aspect of non-physical disability in MS patients, which is still lacking objective biomarkers, so analysis of EP parameters with regards to fatigue in MS seemed worth investigating. We deliberately chose VEP and BAEP as they have not been used in this field so far (in contrast to MEP).

Analysis of VEP showed significantly prolonged latency of the P100 component in the whole group of MS patients as well as in each of the three subgroups, when compared to the controls. Such a finding is common in MS subjects and indicates slowed conduction in the optic tract due to demyelination. It is worth noting that only severely fatigued patients in comparison with non-fatigued ones showed a significantly increased interocular latency difference (relative P100 latency). This parameter tended to correlate (although not significantly) with one of the fatigue measures (FSS/FSS-5), but—apart from visual FS score—did not show significant relationships with other disease-related variables (duration of MS, EDSS or MSSS). Although no correlation was found between relative P100 latency and the result of MFIS (which allows more detailed assessment of fatigue than FSS), it might be interesting to refer VEP results to physical and cognitive aspects of fatigue. Significant differences in P100 amplitude were also asymmetrical but they were found not only between fatigued and non-fatigued subgroups but also between MS patients and controls. The amplitude of VEP components is usually regarded as a more variable and thus less sensitive parameter than latency, so we believe P100 latency deserves more attention in further investigation.

P100 latency is known to increase with age, especially in men. Subgroups I, II and III did not differ significantly as regards age and gender structure, so the influence of demographic factors upon VEP parameters can be neglected.

In the only available study comprising MS patients (i.e., Regan et al. [18]), VEP were used to evaluate the fatigability of the visual pathway. In those patients with MS and glaucoma (but not in parkinsonic ones), the amplitude of P100 increased when additional stimuli were superimposed on the basic pattern of stimulation. Our results seem more consistent with the report of Sobieszczańska et al. [19], who assessed VEP as a measure of fatigability in healthy persons, professionally operating computer terminals. After a few hours of their constant gazing at the computer screen, there was an increase in P100 latency, a decrease in amplitude, and moreover, a decrease in correlation coefficients for the VEP parameters obtained from both hemispheres. Overall, the abnormalities of VEP parameters in our material can be attributed to the impact of MS in general, but the asymmetry of these abnormalities might have been more specific for fatigue.

Unlike the optic tract, the auditory pathway is much less frequently affected by demyelination in the course of MS. In the whole group of our MS patients in comparison with the controls, we only found significantly prolonged interlatencies between I, III and V components of BAEP, which indicate subtle conduction disturbances within the brainstem. On analysis of the subgroups of patients with and without fatigue, these abnormalities appeared to occur only in those with moderate and severe fatigue. It has to be considered that these subgroups also presented with higher level of disability and rate of its progression. The interlatencies of BAEP components indeed showed significant correlations with MSSS but not with any of the fatigue measures. Moreover, the fatigued patients also showed prolonged latency of the V component of BAEP (while non-fatigued ones and the whole MS group did not). This parameter, in turn, did not correlate significantly with the majority of disease-related variables, apart from Brainstem FS. It is worth noting that significant findings in BAEP parameters mostly concerned only one side.

To our knowledge, there have been no reports on BAEP with regards to fatigue in MS patients. Neri et al. [20] and Bianchedi et al. [21] described abnormalities of BAEP (the lack of component I and prolonged interlatencies) in subjects with chronic fatigue syndrome, which occurred only at higher frequencies of auditory stimulation, so were apparently revealed at a greater burden to the auditory pathway.

The relationship between fatigue and other symptoms and signs of neurological deficit remains a disputable matter [1, 2]. In our study, fatigue measures showed significant correlations with EDSS (general degree of disability, although mostly determined by ambulation skills) as well as with visual and brainstem FS scores. Fatigue is a complex phenomenon, not limited to incapability of physical effort due to motor deficit, but also possibly associated with dysfunction of other systems. Thus, VEP and BAEP, as sensitive and objective markers of visual and brainstem pathways (their parameters correlated significantly with corresponding FS scores), might provide measures of other aspects of disability contributing to fatigue.

The asymmetry of EP abnormalities in our study seemed more specifically associated with fatigue than with MS itself. Asymmetrical damage to CNS pathways interferes with the perception and integration of stimuli of particular modality. To compensate for these dysfunctions, some additional areas of the brain may become activated. This corresponds with the concept of fatigue as a result of excessive load of CNS due to dysfunction of specific areas, as is supported by neuroimaging studies involving MS patients with fatigue [3, 22, 23].

To the best of our knowledge, so far there has been no report investigating visual and auditory EP with regards to fatigue in a large and well-defined group of MS patients. Abnormalities of EP in fatigued patients, independent from MS-related variables, may support the hypothesis of disturbed bioelectrical activity due to CNS damage as the background of fatigue, which contradicts the idea of its purely subjective origin. EP parameters seem promising as possible electrophysiological markers of fatigue with the asymmetry of their abnormalities deserving special attention. A limitation of our study is the fact that the assessment of fatigue and EP parameters was performed only once, without re-testing to check for reliability of the results. Considering the common fluctuations of MS symptoms and the variability of EP parameters, we have already planned further study including parallel monitoring of fatigue and EP in the course of the disease to evaluate their relationships in prospective observation.

In conclusion, the parameters of VEP and BAEP undergo significant, mostly asymmetrical changes in MS patients with moderate and severe fatigue. These findings seem to support the hypothesis of neuronal pathways dysfunction as the background of MS fatigue. The role of EP parameters as electrophysiological markers of fatigue seems promising and deserves further investigation.
